# A retrospective, observational, epidemiological study of meningococcal meningitis cases in the UK in relation to the change in smoking legislation

**DOI:** 10.1186/1753-6561-6-S4-P26

**Published:** 2012-07-09

**Authors:** H Preston

**Affiliations:** 1 University of Edinburgh, UK

## Introduction

*Neisseria Meningitidis* is the greatest risk to young adults for fatal meningitis. It has been seen in other studies that smokers carry an increased amount of this bacteria and can be carriers for the infection. The aim of the study is to determine whether the smoking legislation brought into the UK has had an effect on the case numbers of meningococcal meningitis, as smoking in seen as a risk factor.

## Methods

Data was obtained from HPS and HPA to get national figures over a decade spanning the smoking ban legislation.

## Results

Results show that there has not been a dramatic decline in cases of meningococcal meningitis since the introduction of the smoking ban.

## Conclusions

There should be continued surveillance in the future to see if a there is a long term trend with rates of meningococcocal meningitis cases.

